# Successful laparoscopic distal gastrectomy with D2 lymph node dissection preserving the common hepatic artery branched from the left gastric artery for advanced gastric cancer with an Adachi type VI (group 26) vascular anomaly

**DOI:** 10.1186/s40792-016-0182-1

**Published:** 2016-06-03

**Authors:** Hironobu Goto, Takashi Yasuda, Taro Oshikiri, Tatsuya Imanishi, Hironori Yamashita, Masato Oyama, Keitaro Kakinoki, Tadayuki Ohara, Hiroyoshi Sendo, Yasuhiro Fujino, Masahiro Tominaga, Yoshihiro Kakeji

**Affiliations:** Division of Gastroenterological Surgery, Hyogo Cancer Center, 13-70, Kitaoji-cho, Akashi, Hyogo 673-8558 Japan; Division of Gastrointestinal Surgery, Department of Surgery, Graduate School of Medicine, Kobe University, 7-5-2, Kusunoki-cho, Chuo-ku, Kobe, Hyogo 650-0017 Japan

**Keywords:** Advanced gastric cancer, Laparoscopic distal gastrectomy, D2 lymph node dissection, Adachi type VI vascular anomaly

## Abstract

We report a case of successful laparoscopic distal gastrectomy with D2 lymph node dissection preserving the common hepatic artery branched from the left gastric artery for advanced gastric cancer with an Adachi type VI (group 26) vascular anomaly. A 76-year-old female patient was admitted with a diagnosis of advanced gastric cancer at the anterior wall to the lesser curvature of the antrum (cT3N0M0 cStage IIA). Dynamic computed tomography showed the ectopia of the common hepatic artery branched from the left gastric artery. We made a diagnosis of an Adachi type VI (group 26) vascular anomaly and performed the abovementioned operation. In this anomaly pattern, scrupulous attention is required to remove the suprapancreatic lymph nodes because the portal vein is located immediately dorsal to those lymph nodes and is at increased risk for the injury in this situation. The common hepatic artery is branched from the left gastric artery, and the hepatic perfusion from the superior mesenteric artery is not present in group 26. Planning to preserve the artery will improve safety when it is possible oncologically. There were no postoperative complications, and the patient was discharged 9 days after the operation. To our knowledge, the present case is the first reported case of a laparoscopic distal gastrectomy with D2 lymph node dissection with an Adachi type VI (group 26) vascular anomaly. Preoperative diagnostic imaging is very important to prevent surgical complications because the reliable identification of vascular anomaly during an operation is very difficult.

## Background

Gastric cancer is a common malignant disease worldwide. The standard surgical procedure for patients with resectable gastric cancer is gastrectomy with lymph node dissection. Recently, laparoscopic gastrectomy can be performed for not only early gastric cancer but also advanced gastric cancer at some specialized institutions [1]. It is very important that we understand the branching types of the celiac artery through the use of multi-detector row computed tomography because the range of suprapancreatic lymph node dissection differs between D1 and D2 lymph node dissection [2]. Adachi classified branching types of the celiac artery into 6 types and 28 groups [3]. In Adachi type VI, the common hepatic artery is not detected at the superior border of the pancreas, and its frequency is approximately 2 % [3]. Additionally, in group 26, the common hepatic artery is branched from the left gastric artery, and the frequency is approximately 0.4 % [3]. We report a case of advanced gastric cancer with an Adachi type VI (group 26) vascular anomaly that was successfully treated by laparoscopic distal gastrectomy with D2 lymph node dissection preserving the common hepatic artery branched from the left gastric artery. The International Union Against Cancer (UICC) TNM staging system for gastric cancer was used for tumor staging [4]. The lymph node stations were defined according to the definitions of the Japanese Gastric Cancer Association (JGCA) [5].

## Case presentation

A 76-year-old female patient was admitted to our hospital with a gastric cancer identified by gastroduodenal endoscopic examination. No physical abnormalities were observed, and laboratory data, including hematologic and biochemical analyses, revealed no abnormalities.

**Fig. 1:**
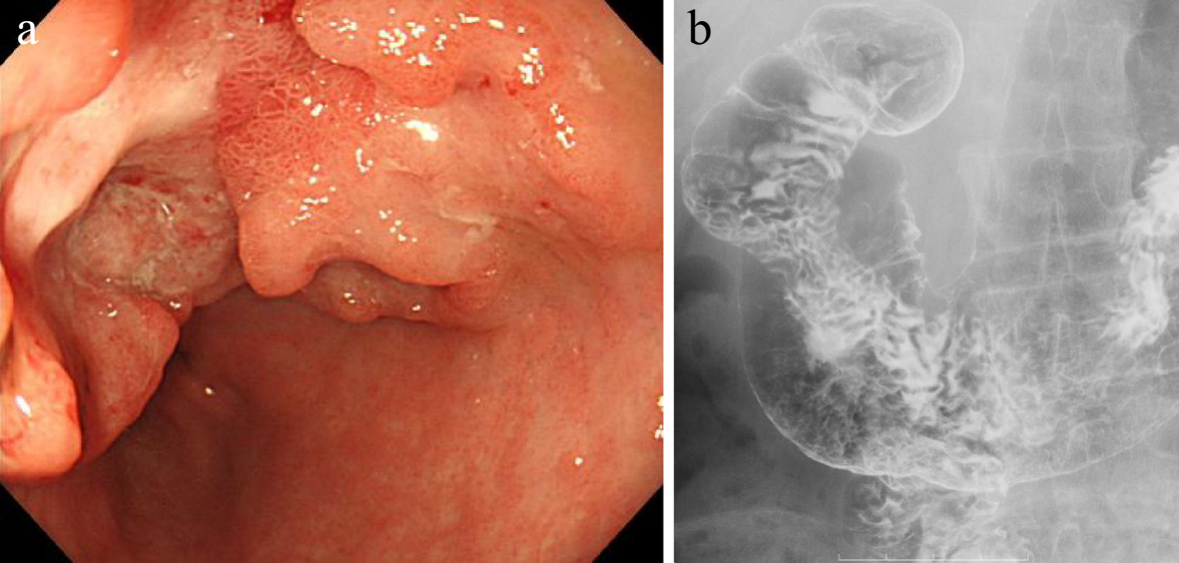
**a**, **b** Findings of the upper gastrointestinal series. A type 3 tumor was shown, with ulceration at the anterior wall to the lesser curvature of the antrum of the stomach

**Fig. 2:**
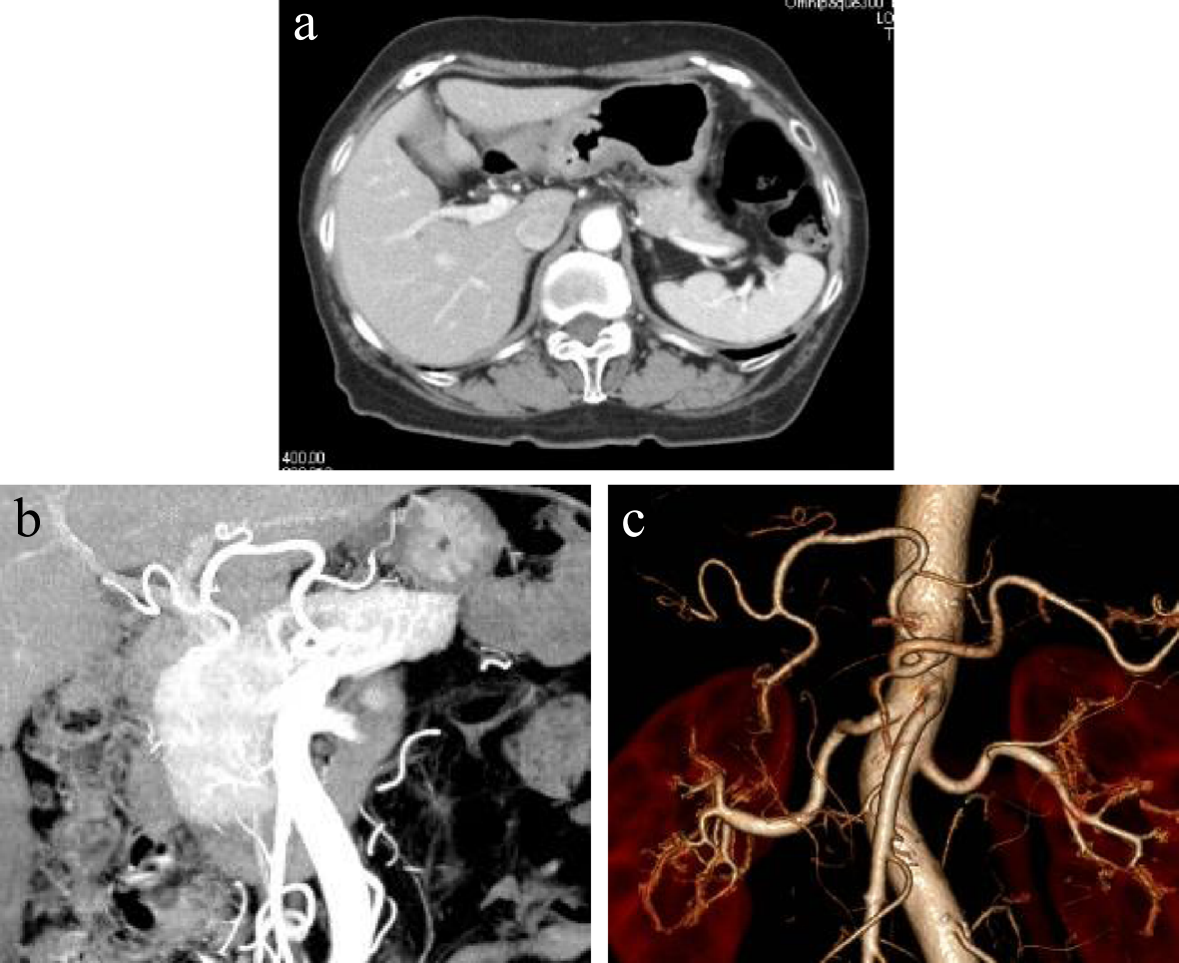
Findings of the enhanced computed tomography scan. **a** Wall thickening of the stomach that was a suspected invasion of the subserosa. **b**, **c**:The dynamic computed tomography and computed tomography angiogram showed the ectopia of the common hepatic artery branched from the left gastric artery

A gastroduodenal endoscopic examination and upper gastrointestinal series revealed the presence of a type 3 gastric tumor (30 mm) at the anterior wall to the lesser curvature of the antrum (Fig. 1a, b). A biopsy of the tumor was performed, and the pathological diagnosis was well-differentiated tubular adenocarcinoma of the stomach. The enhanced computed tomography scan showed wall thickening of the stomach that was a suspected invasion of the subserosa (Fig. 2a). Additionally, there were no lymph node metastasis and no metastasis in other organs, such as the liver and lungs (cT3N0M0 cStage IIA). The dynamic computed tomography and computed tomography angiogram showed the ectopia of the common hepatic artery branched from the left gastric artery, and we diagnosed an Adachi type VI (group 26) vascular anomaly (Fig. 2b, c).

This patient was enrolled in the randomized trial of open and laparoscopic distal gastrectomy with D2 lymph node dissection for locally advanced gastric cancer conducted within the framework of the Japanese Laparoscopic Surgery Study Group (JLSSG 0901 trial) [1]. This case was randomly allocated to the laparoscopic surgery group.

In our institution, the laparoscopic distal gastrectomy was performed using five trocars. The first 12-mm trocar was inserted transumbilically, 12- and 5-mm trocars were inserted above and to the right side of the umbilicus, and the other two 12-mm trocars were inserted above and to the left side of the umbilicus. Carbon dioxide pneumoperitoneum was maintained at 10 mmHg, and lymph node dissection was carried out using an ultrasonically activated device through the four operative trocars.

**Fig. 3:**
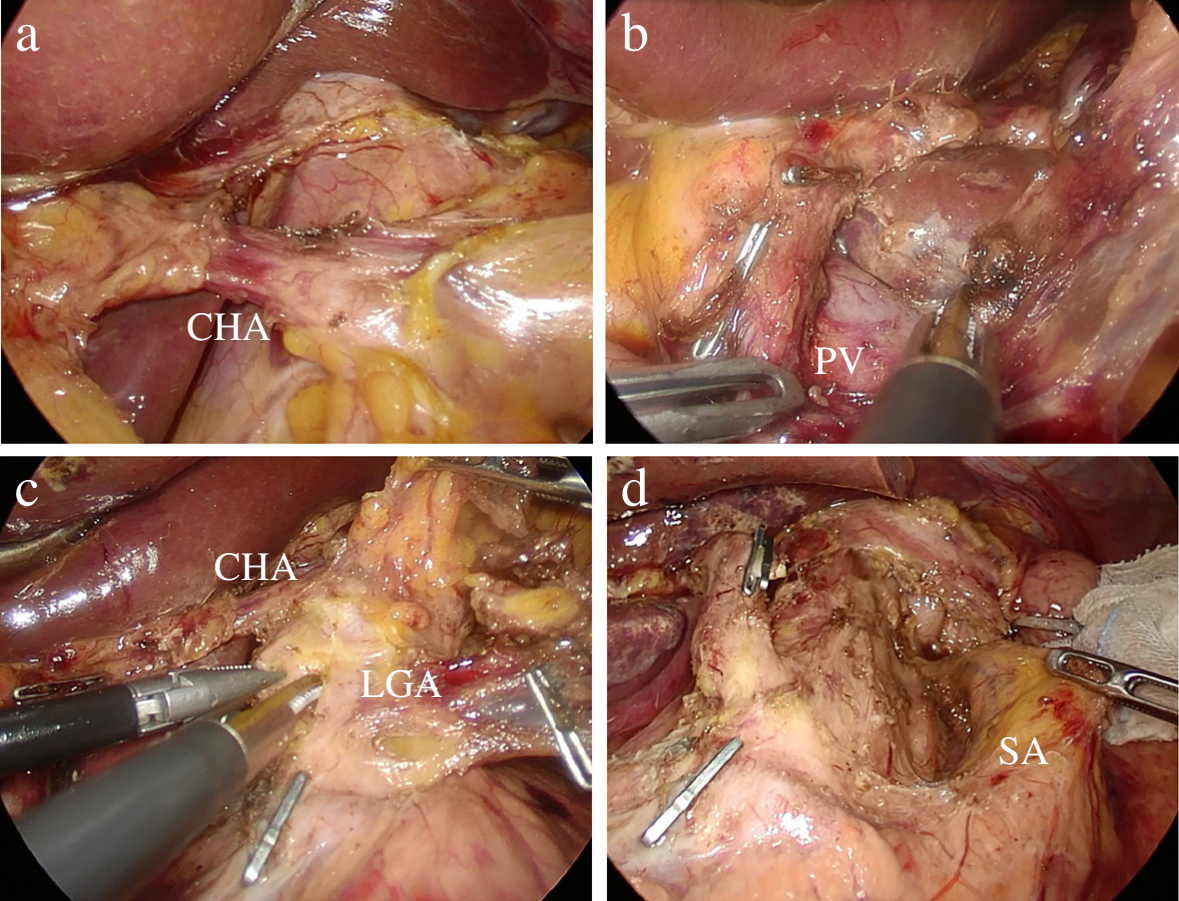
**a** The common hepatic artery of the lesser omentum was encircled. **b** The dissection of station 12a and exposure of the portal vein was performed. **c** The anterior surficial tissue of the left gastric artery was divided like a clamshell door. **d** The lymph node dissection of station 11p along the splenic artery was performed. *CHA* common hepatic artery, *PV* portal vein, *LGA* left gastric artery, *SA* splenic artery

**Fig. 4:**
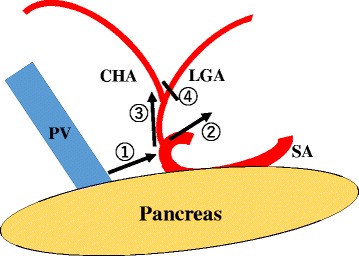
The lymph node dissection around the root of the left gastric artery. *1* The tissue containing suprapancreatic lymph nodes was removed, and the root of the left gastric artery was detected. *2* The space between the left gastric artery and the tissue containing station 11p was separated. *3* The anterior surficial tissue of the left gastric artery was divided like a clamshell door. *4* The artery of a gastric branch from the left gastric artery was divided. *CHA* common hepatic artery, *LGA* left gastric artery, *SA* splenic artery, *PV* portal vein

In intra-abdominal findings, there were no distant metastatic lesions in other organs, such as the liver and peritoneum, and a primary lesion could not be detected from the visible surface of the stomach. Saline was used to irrigate the Pouch of Douglas, and the cytological examination showed no malignancy. Laparoscopic distal gastrectomy with D2 lymph node dissection was performed according to the Japanese Gastric Cancer treatment guidelines (2010) [2]. The right and left greater omentum and lymph nodes were dissected along the gastroepiploic artery and infrapyloric vessels, and the duodenum was transected by a linear stapler. The left lobe of the liver was retraced using the Nathanson liver retractor (Cook Japan, Tokyo, Japan), and the suprapancreatic lymph node dissection was started. The adipose tissue containing the suprapancreatic lymph nodes was stretched and dissected along the outermost layer of the proper hepatic artery due to replacing the common hepatic artery. The lesser omentum was divided, and the common hepatic artery in the lesser omentum was encircled (Fig. 3a). The right gastric artery was divided, and the dissection of station 12a and exposure of the portal vein was performed (Fig. 3b). The lymphatic connection between the dissected tissue and paraaortic lymph nodes was separated, and the tissue containing stations 8a, 9, and 12a was removed along the right diaphragmatic crus. The anterior surficial tissue of the left gastric artery was divided like a clamshell door (Fig. 3c). The fatty tissue containing stations 8a, 9, and 12a was transferred through the posterior of the left gastric and the common hepatic artery, and the gastric branch from the left gastric artery was divided. It was showed the detail of the lymph node dissection around the root of the left gastric artery (Fig. 4). The tissue containing station 11p was freed from Gerota’s fascia, thereby delineating the dorsal area of the station 11p lymph node, and a dissection along the splenic artery was performed (Fig. 3d). Finally, the stomach was divided into 5 cm proximally to the tumor by a linear stapler after the lymph node dissection of the lesser curvature. The location of the tumor was marked with black ink during an endoscopy before the surgery. The excised specimen was removed through the umbilical trocar site using a bag. The Billroth-I reconstruction was performed intracorporeally with a delta-shaped anastomosis [6]. The operation time was 353 min, and the blood loss was 20 g. There were no postoperative complications, and the patient was discharge 9 days after the operation.

The pathological examination revealed a type 3 tumor (35 × 30 mm) in the stomach exhibiting well and moderately differentiated tubular adenocarcinoma with no metastases in 58 harvest lymph nodes (pT2N0M0 pStageIB).

### Discussion

Recently, the number of patients undergoing laparoscopic gastrectomy for gastric cancer has increased [7]. Because many studies reported that laparoscopic distal gastrectomy for patients with early gastric cancer was a minimally invasive procedure, and that long-term outcomes were comparable with open distal gastrectomy, laparoscopic distal gastrectomy for patients with early gastric cancer has been widely accepted [8–11]. Although the results of the multi-institutional randomized phase II trial (JLSSG 0901 trial) demonstrated the technical safety of laparoscopic distal gastrectomy with D2 lymph node dissection for patients with advanced gastric cancer, a phase III trial (JLSSG 0901 trial) which is the extension of the study that compared laparoscopic surgery with open surgery in term of oncological outcome is ongoing [1].

Laparoscopic surgeons can perform a precise operation, because the magnified surgical field is provided by laparoscopic surgery. However, the procedure is potentially disadvantageous because the identification of the anatomy is difficult due to an absence of thigmesthesia. Therefore, the identification of a vascular anomaly prior to surgery is very important for the safety and accuracy of the operation. It has been reported that multi-detector computed tomography is useful preoperatively to plan surgical strategy, thereby optimizing the safety and efficacy of laparoscopic gastrectomy [12]. In gastric surgery, the area of the suprapancreas is one of the most challenging sites; we perform lymph node dissection strictly and carefully because of its various anatomical variants of the vessels.

**Fig. 5:**
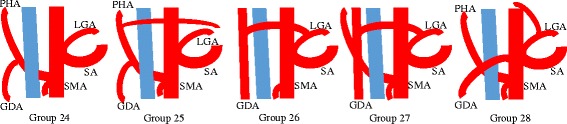
Adachi type VI vascular anomaly. *PHA* proper hepatic artery, *GDA* gastroduodenal artery, *LGA* left gastric artery, *SA* splenic artery, *SMA* superior mesenteric artery

Adachi classified the anatomy of the common hepatic artery, the left gastric artery, and the splenic artery originating from the celiac artery into 6 types and 28 groups [3]. In Adachi type VI, the common hepatic artery is not detected at the superior border of the pancreas, and blood flow to the liver is supplied from the accessory hepatic artery (Fig. 5). In this anomaly pattern, scrupulous attention is required to remove the suprapancreatic lymph nodes because the portal vein is located immediately dorsal to those lymph nodes and is at an increased risk for injury in this situation. The hepatic perfusion from the superior mesenteric artery is present in the Adachi type VI, but not in group 26. Therefore, the accessory hepatic artery in the lesser omentum can be divided. However, it is necessary to understand that the common hepatic artery is branched from the left gastric artery and the hepatic perfusion from the superior mesenteric artery is not present in group 26. Even if the left gastric artery is divided at the root, the hepatic perfusion may be of no consequence because of the blood flow from around the pancreatic head, at least in theory. We should plan to preserve the artery to improve safety when it is possible oncologically. In the present case, we diagnosed no lymph node metastasis preoperatively and removed the lymph nodes preserving the common hepatic artery. When lymph node metastasis is suspected and the artery cannot be preserved, we should confirm that the left gastric artery is clamped and check the color of the liver and pulsation of the proper hepatic artery. According to circumstances, a Doppler ultrasonography of the liver may be very useful.

To our knowledge, this is the first case report on laparoscopic distal gastrectomy with D2 lymph node dissection preserving the common hepatic artery branched from the left gastric artery with an Adachi type VI (group 26) vascular anomaly. Because the laparoscopic surgery is potentially disadvantage due to an absence of thigmesthesia, it is very difficult to detect an Adachi type VI (group 26) vascular anomaly reliably during an operation. Therefore, preoperative diagnostic imaging is very important for the prevention of the surgical complications, thereby minimally invasive surgery can be safely performed as well as open surgery.

## Conclusions

In summary, we reported a successful laparoscopic distal gastrectomy with D2 lymph node dissection preserving the common hepatic artery branched from the left gastric artery for advanced gastric cancer with an Adachi type VI (group 26) vascular anomaly. Special attention should be paid to the vascular anatomy using preoperative diagnostic imaging.

## Consent

Written informed consent was obtained from the patient for publication of this case report and the accompanying images.
